#  Emerging Infectious Diseases Journal: A Time of Transition

**DOI:** 10.3201/eid0801.030102

**Published:** 2002-01

**Authors:** Joseph E. McDade, James M. Hughes

**Figure 1:**
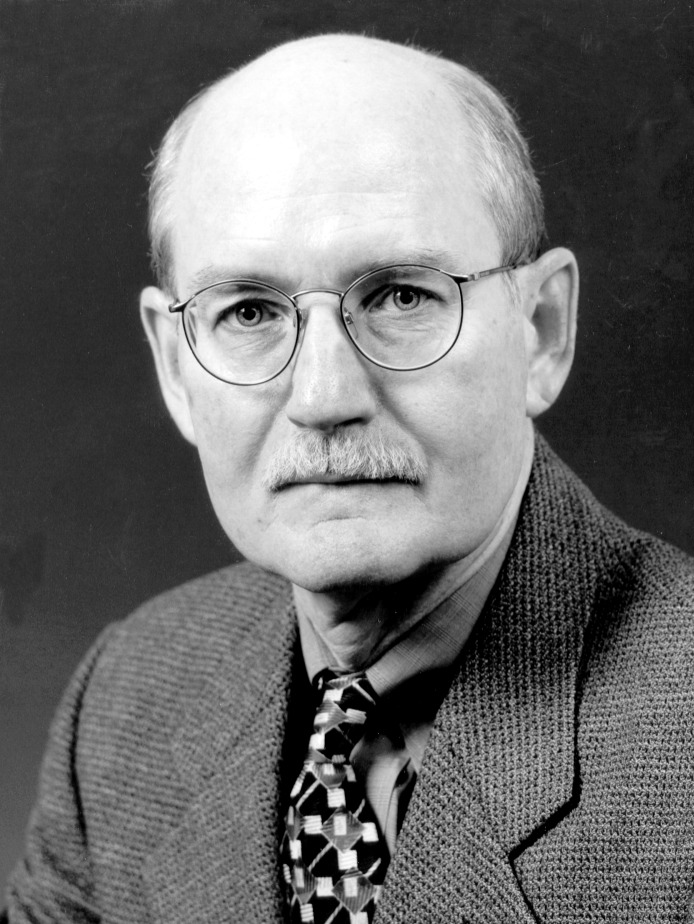
Dr. Joseph McDade Founding Editor, Emerging Infectious Diseases journal.

**Figure 2:**
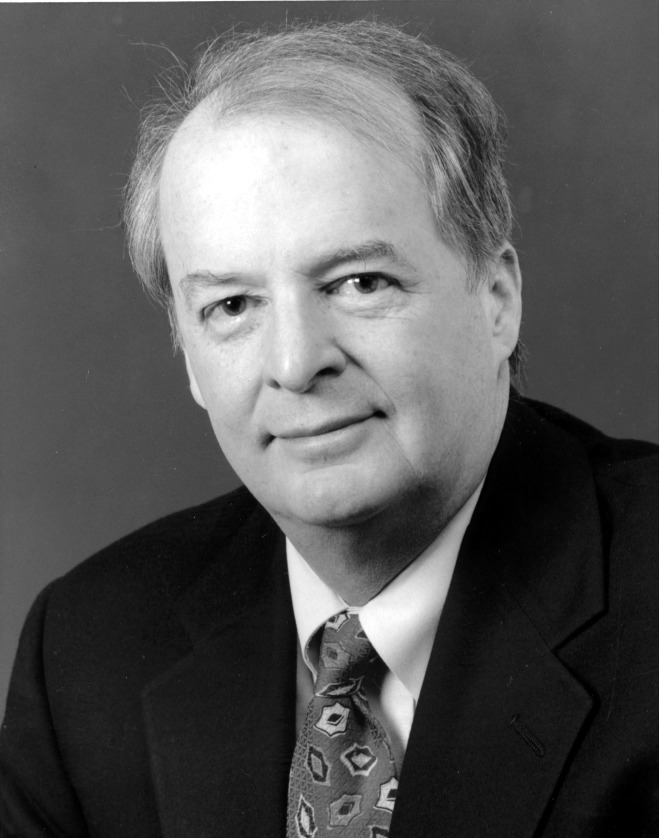
Dr. James Hughes Director, National Center for Infectious Diseases, Centers for Disease Control and Prevention

Change is a constant for Emerging Infectious Diseases. Since its inception in 1995, the journal has undergone many transformations: its content has broadened, its format has become more distinctive, and its frequency of publication has increased, from quarterly to bimonthly in 1999 and now to monthly with this issue. Yet, amidst all the changes, Emerging Infectious Diseases has found its niche in the ranks of scientific journals, serving the needs of professionals in infectious diseases and many related disciplines. In 7 short years, the journal has achieved a high impact factor (#3 of 36 infectious disease journals, ISI Citation Reports, 2000); indexing in Medline, Current Contents, and other major databases; translations into Spanish, Japanese, Chinese, and French; and a circulation of more than 45,000 subscribers to its print and electronic versions. The journal has also provided a new communication channel through which to encourage global investigation and dialogue on the many issues, challenges, and opportunities posed by emerging infectious diseases.

The goals of the journal stem directly from the Centers for Disease Control and Prevention’s (CDC’s) action plan against emerging infections: recognition of new and reemerging infections; understanding of factors involved in disease emergence, prevention, and elimination; and fast and broad dissemination of reliable information on emerging infections around the world. Toward these goals, the journal 1) provides information on factors known to influence emergence: microbial adaptation and change, human demographics and behavior, technology and industry, economic development and land use, international travel and commerce, and the breakdown of public health measures; 2) reports laboratory and epidemiologic findings within a broader public health perspective; 3) provides timely updates of infectious disease trends and research (new methods of detecting, characterizing, or subtyping pathogens; developments in antimicrobial drugs, vaccines, and prevention or elimination programs; case reports); 4) publishes reports of interest to researchers in infectious diseases and related sciences, as well as to public health generalists learning the scientific basis for prevention programs; 5) encourages insightful analysis and commentary, stimulating global interest in and discussion of emerging infectious disease issues; and 6) harnesses electronic technology to expedite and enhance global dissemination of emerging infectious disease information. 

In pursuing these goals, the journal has set a high standard of quality, not only for its peer-reviewed scientific content but also for the readability and accessibility of this content. A modern journal functions in a fast-paced world of information overload, instant access, overburdened scientific audience, and the accidental broader audience created by the World-Wide Web. To fulfill the increasing expectations of an expanding audience that includes scientists in many disciplines, public health generalists, students, and the public, articles are edited for language, communication effectiveness, style, and length. The human aspect of scientific research is always kept in the forefront by the association of scientific research with works of art on the journal’s cover and by “Another Dimension” articles, reminding the reader that, in the end, the purpose of all scientific endeavor is the betterment of humanity and the improvement of the quality of life for all people. 

The journal relies on a broad international authorship base and rigorous independent peer review to provide accurate and reliable scientific information on emerging infections, free of charge, around the globe. The journal draws authors and readers from professionals in infectious diseases and many other scientific disciplines in the United States and abroad, specialists in academia, industry, clinical practice, and public health, as well as economics, demography, and sociology. To expedite dissemination of up-to-the-minute information, from its inception Emerging Infectious Diseases has published articles online ahead of print. 

Since 1995, Emerging Infectious Diseases has published new scientific research, analysis and commentary, policy reviews, and concise synopses on a broad array of infectious disease topics. Coverage has included topics as diverse as *Morbillivirus* in Australia, tuberculosis trends in Japan, antimicrobial resistance in Europe, infectious disease emergence in New Zealand, genomics and bacterial pathogenesis, amphibian population declines, the role of migratory birds in the spread of *West Nile virus*, infections in the health-care setting, bovine spongiform encephalopathy and variant Creutzfeldt-Jakob disease, and bioterrorism.

In 2002, the journal remains at the crossroads of change. Upon my retirement from CDC, D. Peter Drotman, a senior scientist with the National Center for Infectious Diseases, became Interim Editor while a search is conducted for a new Editor-in-Chief. To the role of Interim Editor, Dr. Drotman brings a broad knowledge of infectious diseases, effective leadership, and boundless enthusiasm. A veteran of the successful World Health Organization Smallpox Eradication Programme, he was among the first CDC scientists assigned to investigate cases of what was later to be named AIDS.

In the meantime, infections continue to emerge: new infections resulting from changes or evolution of existing organisms, known infections spreading to new geographic areas or populations, previously unrecognized infections appearing in areas undergoing ecologic transformation, old infections reemerging as a result of antimicrobial resistance in known agents or breakdowns in public health measures. The recent appearance of *West Nile virus* in the United States and the use of *Bacillus anthracis* in recent bioterrorism attacks are reminders of the need for vigilance and the undiminished potential for global dissemination of infectious agents.

As a monthly journal in 2002, Emerging Infectious Diseases will continue to track and analyze infectious disease trends and to encourage investigation and timely communication of emerging threats and critical related issues around the world. Advances in electronic publishing allow unprecedented speed in disseminating public health information and create new opportunities for innovation and improvements in communication. Electronic submission and peer review of manuscripts, online publication ahead of print, online-only publication, and convenient links to other sources of biomedical information are only the beginning.

